# Frequencies of polymorphisms associated with BSE resistance differ significantly between *Bos taurus*, *Bos indicus*, and composite cattle

**DOI:** 10.1186/1746-6148-4-36

**Published:** 2008-09-22

**Authors:** Brian W Brunelle, Justin J Greenlee, Christopher M Seabury, Charles E Brown, Eric M Nicholson

**Affiliations:** 1 Virus and Prion Diseases of Livestock Research Unit, National Animal Disease Center, USDA, Agricultural Research Service, Ames, IA 50010 USA; 2 Department of Veterinary Pathobiology, College of Veterinary Medicine, Texas A&M University, College Station, TX 77843-4467 USA; 3 ABS Global Inc., Deforest, WI 53532 USA; 4 Pre-Harvest Food Safety and Enteric Diseases Research Unit, National Animal Disease Center, USDA, Agricultural Research Service, Ames, IA 50010 USA

## Abstract

**Background:**

Transmissible spongiform encephalopathies (TSEs) are neurodegenerative diseases that affect several mammalian species. At least three factors related to the host prion protein are known to modulate susceptibility or resistance to a TSE: amino acid sequence, atypical number of octapeptide repeats, and expression level. These factors have been extensively studied in breeds of *Bos taurus *cattle in relation to classical bovine spongiform encephalopathy (BSE). However, little is currently known about these factors in *Bos indicus *purebred or *B. indicus *× *B. taurus *composite cattle. The goal of our study was to establish the frequency of markers associated with enhanced susceptibility or resistance to classical BSE in *B. indicus *purebred and composite cattle.

**Results:**

No novel or TSE-associated *PRNP*-encoded amino acid polymorphisms were observed for *B. indicus *purebred and composite cattle, and all had the typical number of octapeptide repeats. However, differences were observed in the frequencies of the 23-bp and 12-bp insertion/deletion (indel) polymorphisms associated with two bovine *PRNP *transcription regulatory sites. Compared to *B. taurus*, *B. indicus *purebred and composite cattle had a significantly lower frequency of 23-bp insertion alleles and homozygous genotypes. Conversely, *B. indicus *purebred cattle had a significantly higher frequency of 12-bp insertion alleles and homozygous genotypes in relation to both *B. taurus *and composite cattle. The origin of these disparities can be attributed to a significantly different haplotype structure within each species.

**Conclusion:**

The frequencies of the 23-bp and 12-bp indels were significantly different between *B. indicus *and *B. taurus *cattle. No other known or potential risk factors were detected for the *B. indicus *purebred and composite cattle. To date, no consensus exists regarding which bovine *PRNP *indel region is more influential with respect to classical BSE. Should one particular indel region and associated genotypes prove more influential with respect to the incidence of classical BSE, differences regarding overall susceptibility and resistance for *B. indicus *and *B. taurus *cattle may be elucidated.

## Background

Transmissible spongiform encephalopathies (TSEs) are a class of neurodegenerative diseases that affect various mammals, including cattle, sheep, mink, cervids, and humans. They are caused by abnormally folded prion proteins that induce the conversion of the normal and non-infectious cellular form of the host prion protein (PrP^C^) into the abnormal and infectious form (PrP^Sc^) [1]. Susceptibility or resistance to a TSE can be influenced by several factors of the host prion protein, such as specific amino acid polymorphisms, the number of octapeptide repeats present, and prion protein expression levels. These 3 factors are all relevant to prion biology in cattle.

Bovine spongiform encephalopathy (BSE) is a TSE of cattle. Based upon Western blot and *in vivo *analysis, BSE can be differentiated into two strains, classical and atypical BSE [2-4]. Although amino acid differences in the prion protein are a major component in susceptibility and resistance to TSE disease in humans [5] and sheep [6], they are not associated with classical BSE cases in cattle. However, the prion protein gene (*PRNP*) for the 2006 US atypical BSE case encoded an amino acid change in one allele at bovine codon 211 (Glutamic Acid → Lysine; E211K) [7]. This change is analogous to the human E200K amino acid replacement, which is associated with the leading cause of heritable TSE disease in humans [5]. To date, the E211K change has been reported in only two bovine samples, the atypical BSE-positive cow [7] and its only known living offspring [8].

The octapeptide repeat region is a series of amino acid repeats near the N-terminal portion of PrP^C^, has been implicated in binding divalent cations, and may affect the structure and function of the prion protein [9]. Humans, sheep, and cervids (deer) normally possess 5 octapeptide repeats, while cattle typically have 5 or 6 repeats [10,11]. The presence of extra repeats encoded within the octapeptide region is correlated with an increase in TSE susceptibility, as has been observed in humans that possess more than 5 octapeptide repeats [12,13]. Additionally, transgenic mice expressing bovine PrP^C ^containing 7 or 10 repeats are also more susceptible when challenged with BSE [14,15]. Of the breeds tested to date, only Brown Swiss cattle are known to encode 7 octapeptide repeats [16,17], and they have been reported to be more susceptible to BSE than other cattle breeds [18,19]. These data suggest that bovine PrP^C ^containing 7 or more octapeptide repeats may enhance susceptibility to BSE.

In addition to qualitative changes in the mammalian prion protein itself, the level of mammalian PrP^C ^expression is also known to influence susceptibility or resistance to a TSE disease. Over-expression of PrP^C ^in transgenic mice challenged with a TSE resulted in shorter incubation periods as compared to wild type mice [20,21]. Conversely, transgenic mice possessing one functional *PRNP *allele had decreased expression levels of PrP^C^, which led to a longer incubation time after a TSE inoculation [22]. Mice lacking functional *PRNP *alleles (*Prnp*^0/0^) were resistant to TSE challenge [23]. In cattle, two non-coding polymorphisms have been associated with PrP^C ^expression levels [24,25]. The first is a 23-bp deletion within the promoter region that removes a binding site for the RP58 repressor protein, and the second is a 12-bp deletion within intron 1 that removes a SP1 transcription factor binding site [26]. Cattle possessing these deletions, and therefore lacking binding sites for their respective regulatory elements, have been reported to be more susceptible to classical BSE [24,26]. These polymorphisms do not influence resistance to atypical BSE [27,28].

To date, most analyses of cattle populations for these specific BSE susceptibility factors have focused on breeds derived from *Bos taurus*. However, few relevant studies currently exist for *Bos indicus *or *B. indicus *× *B. taurus *composite cattle. Since *B. indicus *purebred and composite cattle are dispersed throughout the world, we elected to determine the frequencies of known genetic factors associated with BSE susceptibility and resistance in a diverse sample intended to represent the global population. In this report, we provide a detailed comparative analysis of the 23-bp promoter region, 12-bp intron region, and relevant *PRNP *polymorphisms for *B. indicus*, *B. taurus*, and *B. indicus *× *B. taurus *composite cattle. Differences in the frequencies of these established risk factors may also elucidate differences in overall resistance and/or susceptibility to classical BSE between the cattle groups investigated.

## Results

### PRNP indel allele and genotype frequencies

Allele and genotype frequencies for the 23-bp and 12-bp indel regions were compared between *B. indicus*, *B. taurus*, and *B. indicus *× *B. taurus *composite cattle (Table 1). Significant differences were observed in the distribution of alleles and genotypes between *B. indicus *and *B. taurus *cattle with respect to both regions (P < 0.01). However, composite cattle differed from *B. taurus *cattle only for the 23-bp indel (P < 0.01) and from *B. indicus *cattle only for the 12-bp indel (P < 0.01). Interestingly, the *B. indicus *cattle had a significantly lower frequency of the 23-bp promoter insertion allele as compared to *B. taurus*, but had a significantly higher frequency of the 12-bp insertion allele within intron 1. These data are highlighted by the differences at both loci for the homozygous insertion genotypes, which are associated with enhanced putative resistance to classical BSE. For the 23-bp homozygous insertion, the frequency observed in *B. taurus *(14%) was much higher than that observed in either *B. indicus *(2%) or composite (3%) cattle, whereas the frequency of the 12-bp homozygous insertion was much higher in *B. indicus *(76%) as compared to either *B. taurus *(20%) or composite (18%) cattle. This disparity is explained by the indel haplotype assignments and corresponding frequencies.

**Table 1:** Allele, genotype, and haplotype frequencies for the 23-bp and 12-bp insertion/deletion polymorphism


**Allele**							

	Frequencies				P-value		
			
23-bp	n	+	-		*B. indicus*	Composite	*B. taurus*

*B. indicus*^a^	116	0.12	0.88		--------	0.1553	<0.0001
Composite^b^	76	0.20	0.80			--------	0.0021
*B. taurus*^c^	4552	0.38	0.62				--------
							
12-bp	n	+	-		*B. indicus*	Composite	*B. taurus*

*B. indicus*^a^	116	0.87	0.13		--------	<0.0001	<0.0001
Composite^b^	76	0.47	0.53			--------	0.7030
*B. taurus*^c^	4564	0.45	0.55				--------

**Genotype**							

	Frequencies				P-value		
		
23-bp	n	+/+	+/-	-/-	*B. indicus*	Composite	*B. taurus*

*B. indicus*^a^	58	0.02	0.21	0.78	--------	0.3061	<0.0001
Composite^b^	38	0.03	0.34	0.63		--------	0.0049
*B. taurus*^c^	2276	0.14	0.48	0.39			--------

12-bp	n	+/+	+/-	-/-	*B. indicus*	Composite	*B. taurus*

*B. indicus*^a^	58	0.76	0.22	0.02	--------	<0.0001	<0.0001
Composite^b^	38	0.18	0.58	0.24		--------	0.4235
*B. taurus*^c^	2282	0.20	0.48	0.32			--------

**Haplotype**							

	Frequencies				P-value		
		
23-12-bp	n	++	-+	--	*B. indicus*	Composite	*B. taurus*

*B. indicus*^a^	116	0.12	0.75	0.13	--------	<0.0001	<0.0001
Composite^b^	76	0.20	0.28	0.53		--------	<0.0001
*B. taurus*^d^	3604	0.40	0.08	0.53			--------

Frequencies and statistical comparisons for the 23-bp and 12-bp alleles, genotypes, and haplotypes in *B. indicus*, *B. taurus*, and *B. indicus *× *B. taurus *composite cattle. Allele frequencies were compared using Fisher's exact test, and genotype and haplotype frequencies were compared using the Chi-square test. Superscript indicates origin of data; complete details can be found in the Methods section and Additional files 1, 2, 3.

^a ^This study, [17]; ^b ^This study, [17]; ^c ^This study, [17,24,33-36,41]; ^d ^This study, [17,24,33,35,36,41]

### PRNP indel haplotype frequencies

Three 23-bp and 12-bp haplotype combinations occur in *B. taurus*, *B. indicus*, and composite cattle: 1) 23-bp insertion-12-bp insertion, 2) 23-bp deletion-12-bp insertion, and 3) 23-bp deletion-12-bp deletion. Haplotype frequencies were different between all three cattle populations (P < 0.01; Table 1). It should be noted that the 23-bp deletion-12-bp insertion is the minor haplotype in *B. taurus *(8%), but it is the major haplotype *B. indicus *(75%).

### PRNP haplotype analysis

The bovine *PRNP *haplotype structure was analyzed for the concatenated 23-bp indel, 12-bp indel, and coding sequence polymorphisms. A total of 41 haplotypes were established among *B. taurus*, *B. indicus*, and composite cattle. For clarity, only the 16 haplotypes with a frequency above 0.02 are shown in Table 2. These 16 haplotypes represent greater than 97% of the *B. taurus *and *B. indicus *cattle and more than 92% of the composite cattle. Haplotypes #9 and #10 were the two most frequent haplotypes among *B. indicus*, comprising 47% of the haplotypes in this group, but they accounted for less than 2% in *B. taurus*. Similarly, haplotypes #3 and #11 represented 78% of *B. taurus *haplotypes, but only 17% in *B. indicus*. Interestingly, haplotype #2 accounted for 17% of the haplotypes among composite cattle, but it was rare in both *B. taurus *and *B. indicus *cattle. These results highlight the 23 and 12-bp indel frequency disparities and species-specific coding region SNPs between *B. taurus *and *B. indicus *cattle.

**Table 2:** Haplotypes and their respective frequencies for *B. indicus*, *B. taurus*, and composite cattle


	23-bp	12-bp	69	75	108	126	# rep	234	339	461	555	576	630	675	678	*B. indicus*	Composite	*B. taurus*

1	-	-	C	G	T	A	6	A	C	G	C	C	C	C	T	----	0.04	0.06
2	-	-	C	G	T	A	6	G	T	G	C	C	C	C	T	0.01	0.17	0.02
3	-	-	C	G	T	A	6	G	C	G	C	C	C	C	T	0.12	0.21	0.40
4	-	-	C	G	T	A	6	G	C	G	C	T	C	C	T	----	0.04	0.04
5	-	+	C	G	T	A	5	--	C	G	C	C	C	C	T	----	0.03	0.03
6	-	+	C	G	T	A	5	--	C	G	C	C	C	C	C	0.02	0.01	----
7	-	+	C	G	T	A	6	A	C	G	T	C	C	C	T	0.10	----	----
8	-	+	C	G	T	A	6	A	C	G	T	C	T	C	T	0.14	0.05	----
9	-	+	C	G	T	A	6	G	C	G	C	C	C	C	T	0.30	0.12	0.01
10	-	+	T	G	T	A	6	G	C	G	C	C	C	C	T	0.17	0.03	<0.01
11	+	+	C	G	T	A	6	A	C	G	C	C	C	C	T	0.05	0.13	0.38
12	+	+	C	G	T	A	6	G	T	G	C	C	C	C	T	----	0.03	0.01
13	+	+	C	G	T	A	6	G	C	G	C	C	C	C	T	----	0.03	0.02
14	+	+	C	G	A	G	6	A	C	A	C	C	C	T	C	0.03	----	----
15	+	+	C	A	A	G	6	G	C	A	C	C	C	C	C	0.03	0.01	----
16	+	+	T	G	T	A	6	G	C	G	C	C	C	C	T	----	0.03	<0.01

Haplotype positions are the 23-bp and 12-bp insertions (+) or deletions (-), nucleotide position in the *PRNP *coding sequence, and the number of octapeptide repeats (#rep). Frequencies of each haplotype in *B. indicus*, *B. taurus*, and composite cattle are listed.

### PRNP coding region

The *PRNP *coding region sequences were compared between *B. indicus*, *B. taurus*, and composite cattle. There were a total of 30 single nucleotide polymorphisms (SNPs), all of which have been reported previously [29], and none of which led to a lysine replacement at codon 211 (E211K). Of the 30 SNPs detected, 5 were found in both *B. indicus *and *B. taurus *cattle, 8 were specific to *B. indicus*, and 17 were specific to *B. taurus *(Table 3). However, 11 of the 17 SNPs in *B. taurus *were only detected in Brown Swiss cattle. The SNP at nucleotide 461 was the only polymorphism that led to an amino acid change (S154N) and was found in *B. indicus *purebred and composite cattle. To date, the S154N change has not been found to be associated with BSE and is not analogous to a TSE-associated polymorphism in another species. Every *B. indicus *and composite sample possessed 5 or 6 octapeptide repeats. The 5 octapeptide repeat allele occurred 51 times in this data set, and 44 of these alleles (86%) were part of the 23-bp deletion-12-bp insertion haplotype. This is significantly different (P < 0.01) than the 6 octapeptide repeat allele, where the 23-bp deletion-12-bp insertion haplotype was only present 161 times in 1343 alleles (12%).

**Table 3:** Shared and species-specific single nucleotide polymorphisms


Species	Single nucleotide polymorphism location								

*B. indicus*	**75**	**108**	**126**	**461**	555	630	675^a^	**678**	
									
*B. taurus*	57^b^	183^c^	189^c^	195^c^	207^c^	210	231^c^	237^c^	255^c^
	261^c^	267^c^	270^c^	294^d^	315^b^	327^c^	378^b^	534^e^	
									
Both species	**69**	**234**	**339**	405	**576**				

Distribution of single nucleotide polymorphisms (SNPs) observed in only *B. indicus *samples, only *B. taurus *samples, or both. Polymorphisms at positions observed in *B. indicus *× *B. taurus *composite samples are in bold and underlined. Polymorphisms observed in only one breed (and therefore not necessarily representative of SNPs in the species) are noted with superscript.

^a^Brahman, ^b^Gelbvieh, ^c^Brown Swiss, ^d^Blonde D'Aquitaine, ^e^Charolais

## Discussion

This study assessed the prevalence of specific BSE-associated factors in *B. indicus *purebred and composite cattle, which were then compared to frequencies observed in *B. taurus *cattle. Through *PRNP *sequence analysis, we surveyed cattle for the presence of an E211K amino acid replacement, as well as the presence of 7 or more octapeptide repeats. In addition, we determined the frequencies of the 23-bp and 12-bp indel regions associated with bovine *PRNP *transcriptional regulation.

None of the *PRNP *alleles for the *B. indicus *samples evaluated in this study exhibited an E211K amino acid replacement or any novel coding region polymorphism. To date, the E211K change has been reported in only two bovine samples, the 2006 Alabama atypical BSE case [7] and its only known living offspring [8]. The affected animal was a composite (*B. taurus *× *B. indicus*), but because no parental information is currently available, it is unknown whether the corresponding nucleotide change was inherited or the result of spontaneous mutation. If it was inherited, then the E211K allele may have originated in either a *B. taurus *ancestor or a *B. indicus *ancestor. Unfortunately, the data presented here cannot facilitate a species level assignment, as the *PRNP *coding sequence of the 2006 Alabama case did not possess any species-specific polymorphisms. This particular animal was determined to possess one haplotype with a 23 and 12-bp insertion, and the other with a 23 and 12-bp deletion [27]. These 2 haplotypes occur in 92% of *B. taurus*, but only in 25% *B. indicus *cattle (Table 1), as estimated by our analyses. Unless more information becomes available, it cannot be determined where the E211K replacement may have originated.

No *B. indicus *sample had an octapeptide region containing more than 6 repeats. Notably, humans are the only TSE-susceptible mammal besides the Brown Swiss breed of *B. taurus *cattle for which additional octapeptide repeats have been observed. Interestingly, a transgenic mouse model expressing bovine PrP^C ^with 1 extra repeat was more susceptible to BSE challenge than a transgenic mouse with the normal number of repeats, but did not develop a spontaneous prion disease [14]. However, a transgenic mouse expressing a bovine *PRNP *gene encoding 4 additional repeats did in fact develop a spontaneous prion disease [15]. While cattle with 1 additional octapeptide repeat may have an enhanced risk for classical BSE only if exposed to infected material, the appearance of *PRNP *genes encoding extra octapeptide repeats in any cattle breed may be cause for concern.

The incidence of E211K as well as octapeptide regions with 7 repeats among cattle does not provide a species-level explanation for potential differences in susceptibility to BSE among *B. taurus *and *B. indicus *cattle. Therefore, only the 23-bp and 12-bp indel regions seem pertinent in these populations because both of these bovine *PRNP *sequence regions have been shown to influence transcription levels of PrP^C^. The *B. indicus *purebred and composite cattle had a very low frequency of the 23-bp insertion as compared to *B. taurus*, while only *B. indicus *purebred cattle had a high frequency of the 12-bp insertion. To date, no consensus has emerged regarding whether one of these bovine *PRNP *regions is more influential than the other with respect to classical BSE resistance in cattle. Originally, only the 23-bp region was found to be significantly associated with (classical) BSE resistance [26]. Using a reporter gene assay, it was later concluded that the 23-bp indel region was the most relevant locus, as the only constructs that lowered expression levels were those containing the 23-bp insertion [25]. In contrast, other reports indicate the 12-bp indel is more relevant both statistically [24] and in a reporter gene assay [30]. The discrepancy between the significance of these two regions with respect to resistance or susceptibility to classical BSE may be influenced by 3 or more factors. First, the 23-bp and 12-bp regions are physically linked (~2-Kbp apart). Therefore, recombination is most likely rare given the small distance separating the two indel polymorphisms. Moreover, high levels of linkage disequilibrium have been detected for genetic variation within the bovine *PRNP *promoter and intron 1 [31]. Secondarily, the 23-bp insertion and 12-bp deletion haplotype is absent among cattle surveyed to date, thereby creating an equal-to-greater overall frequency of 12-bp insertions as compared to the frequency spectrum of 23-bp insertions. More specifically, twice as many haplotypes (n = 12) contribute to the overall frequency of the 12-bp intron 1 insertion as those contributing to the frequency of the 23-bp insertion (n = 6; Table 2). This may inevitably bias indel association studies. Lastly, species specific allelic variation associated with the genetic backgrounds of *B. taurus *and *B. indicus *may differentially interact with the 23-bp promoter and 12-bp intron 1 *PRNP *polymorphisms, perhaps making each polymorphism more or less relevant in a particular bovine species. On the basis of indel genotype alone, if it is ultimately concluded that the 23-bp insertion has a greater influence than the 12-bp insertion with respect to resistance to classical BSE in cattle following exposure to infected material, *B. indicus *purebred and composite cattle would be at greater risk than *B. taurus *cattle. Conversely, if the 12-bp insertion were to modulate a greater level of resistance to BSE, then *B. indicus *cattle would be at a lower risk than *B. taurus *and composite cattle.

## Conclusion

We determined the frequencies of known genetic factors associated with differential susceptibility to BSE in *B. indicus *purebred and *B. indicus *× *B. taurus *composite cattle, as compared to *B. taurus *purebred cattle. No deviations from the expected numbers of octapeptide repeats were detected for *B. indicus *purebred and composite cattle. Likewise, the E211K substitution was not detected within the *PRNP *coding sequences for cattle investigated herein. However, a significant difference was detected for a comparison of the 23-bp and 12-bp indel genotype frequencies between *B. indicus *and *B. taurus *cattle. The origin of this result could be attributed to significant differences in haplotype frequencies among *B. indicus*, *B. taurus*, and composite cattle. Currently, it is unknown which bovine *PRNP *region (23-bp promoter; 12-bp intron 1), if either, may be more important with respect to differential susceptibility to classical BSE in cattle following exposure to the etiologic agent. Should either the 23-bp promoter region or the 12-bp intron 1 region of the bovine *PRNP *prove more biologically relevant to the manifestation of disease, substantial heritable differences in overall susceptibility or resistance to classical BSE may exist between *B. indicus *and *B. taurus *cattle.

## Methods

### Samples

Samples utilized herein were derived from a composite of resources that included DNA, semen, and previously published data. Semen samples from the following 77 unrelated *B. indicus*, *B. taurus*, and composite cattle were provided by ABS Global Inc.: ***B. indicus***: Brahman (26), Nelore (6), Gir (12), Guzerat (1), Tabapua (1); **Composite: **Santa Gertrudis (7), Brangus (10); ***B. taurus***: Shorthorn (14). DNA samples were available from 15 additional unrelated sires: ***B. indicus***: Brahman (3), Nelore (8); **Composite: **Brangus (4). The remaining samples were obtained from the literature, and the breed, number of samples, and citations are as follows: ***B. indicus***: Brahman (1); **Composite: **Beefmaster (4), Braford (4), Brahmousin (2), Brangus (2), Santa Gertrudis (2), Simbrah (3);***B. taurus***: Angus (4), Belgian Blue (4), Blonde D'Aquitaine (5), Braunvieh (5), Charolais (5), Corriente (1), Gelbvieh (4), Hereford (3), Maine Anjou (4), Murray Gray (2), Normande (1), Red Angus (4), Red Poll (1), Salers (3), Scottish Highland (1), Senepol (2), Shorthorn (5), Simmental (8), Tarentaise (1), Texas Longhorn (4), White Park (1) [17,29]; U.S. Holstein (690) [32]; U.K. Holstein (276) [24]; German Holstein (80), German Fleckvieh (60), German Brown (41), Swiss Brown (103), Swiss Scharzfleck (26), Swiss Simmental × Red Holstein (121) [33]; Japanese Holstein (278), Japanese Black (186) [34]; Polish Holstein-Friesian (281) [35]; Korean Holstein (52) [36].

### Genotyping and sequencing

DNA was extracted, amplified, and analyzed as previously described [27]. Briefly, DNA was isolated from semen using the High Pure PCR Template Preparation Kit (Roche Applied Science, Indianapolis, IN). Primer pairs were used to amplify, *via *PCR, a 130 or 153-bp region surrounding the 23-bp promoter indel, a 190 or 202-bp region capturing the12-bp intron 1 indel, and a 986-bp region encompassing the *PRNP *coding region in cattle. Genotypes were distinguished based on PCR product size using a 4% NuSieve gel (Cambrex, Rockland, ME). The *PRNP *coding region was sequenced, and the results were submitted to GenBank (EU564437–EU564528). Frequencies of the 23-bp and 12-bp alleles, genotypes, and haplotypes for each breed are listed in Additional files 1, 2 and 3.

### Haplotype analysis

Unphased genotypes were tested for deviation from Hardy-Weinberg Equilibrium (HWE) using the exact test [37] in conjunction with the online software Genepop  with a cutoff of 0.01. A few alleles were below the cutoff in one of the three cattle groups (*B. indicus*, *B. taurus*, or composite). However, haplotype reconstruction both with and without these alleles proved to be equivalent, so they were kept in the data set for comparative purposes. It should be noted, however, that the samples used in this study violate HWE, as they are not a result of random mating. Nevertheless, violation of the random mating assumption is not known to prevent accurate bovine *PRNP *haplotype reconstructions [31]. Haplotype phases were inferred using a Bayesian statistical approach implemented within the program PHASE 2.1 [38,39]. Haplotype phases that were previously established by cloning and sequencing were designated as such in the raw data, and the octapeptide region was considered a multi-allelic locus since 4–7 repeats have been observed in cattle [29]. Only allele frequencies above 0.10 were used in order to maximize the overall accuracy of the haplotype reconstruction. Analysis was performed using 100 iterations of the data, with 10 additional iterations performed on the final run of the algorithm.

### Statistical analysis

Statistical analyses were performed using GraphPad Prism 4 (Graphpad Software Inc, San Diego, CA). Fisher's exact test [40] was used to test for differences between allele frequencies, and the Chi-square test was used to test for differences between genotype frequencies, as well as between haplotype frequencies (Table 1). Differences between octapeptide repeat allele frequencies and haplotype frequencies were calculated using Fisher's exact test. For all comparisons, *P *≤ 0.05 was considered statistically significant. Using the Bonferroni correction for multiple significance tests for the allele, genotype, and haplotype analyses (k = 15), only the 23-bp genotype test between *B. taurus *and composite cattle was no longer considered significantly different (data not shown).

## Authors' contributions

BWB, EMN, and JJG designed the study. BWB conducted the molecular analyses. BWB and CMS conducted statistical analyses. CMS, JJG, and CEB provided samples. BWB, EMN, and CMS wrote the manuscript. All authors read and approved the manuscript.

## Supplementary Material

Additional file 1 **Allele frequencies for the 23-bp and 12-bp insertion/deletion polymorphism for each breed**. The 23-bp and 12-bp insertion (+) and deletion (-) allele frequencies are listed for each breed of *B. indicus*, *B. taurus*, and *B. indicus *× *B. taurus *composite cattle. Click here for file

Additional file 2 **Genotype frequencies for the 23-bp and 12-bp insertion/deletion polymorphism for each breed**. The 23-bp and 12-bp homozygous insertion (+/+), heterozygous (+/-), and homozygous deletion (-/-) genotype frequencies fro each breed of *B. indicus*, *B. taurus*, and *B. indicus *× *B. taurus *composite cattle. Click here for file

Additional file 3 **Haplotype frequencies for the 23-bp and 12-bp insertion/deletion polymorphism for each breed**. The 23-bp insertion-12-bp insertion (++), 23-bp deletion-12-bp insertion (-+), and 23-bp deletion-12-bp deletion (--) haplotype frequencies for each breed of *B. indicus*, *B. taurus*, and *B. indicus *× *B. taurus *composite cattle. Click here for file
