# Supplementary material for: Frequencies of polymorphisms associated with BSE resistance differ significantly between Bos taurus, Bos indicus, and composite cattle
Source: BMC Vet Res. 2008 Sep 22;4:36. doi: 10.1186/1746-6148-4-36 (PMC2569919; doi:10.1186/1746-6148-4-36)
Supplement: Additional file 1 — Allele frequencies for the 23-bp and 12-bp insertion/deletion polymorphism for each breed. The 23-bp and 12-bp insertion (+) and deletion (-) allele frequencies are listed for each breed of B. indicus, B. taurus, and B. indicus × B. taurus composite cattle. [file 1746-6148-4-36-S1.pdf]

Additional Table 1 - Allele frequencies for the 23-bp and 12-bp insertion/deletion polymorphism for each breed

The 23-bp and 12-bp insertion (+) and deletion (-) allele frequencies are listed for each breed of *B. indicus*, *B. taurus*, and *B. indicus* x *B. taurus* composite cattle.

	23 bp			12 bp		
Breed	n	+	-	n	+	-
B. indicus						
Brahman[a,b]	60	0.18	0.82	60	0.95	0.05
Nelore[a]	28	0.00	1.00	28	0.93	0.07
Gir[a]	24	0.13	0.88	24	0.63	0.38
Guzerat[a]	2	0.00	1.00	2	0.50	0.50
Tabapua[a]	2	0.00	1.00	2	1.00	0.00
Total B. indicus	116	0.12	0.88	116	0.87	0.13
Crossbred						
Santa Gertrudis[a,b]	18	0.17	0.83	18	0.33	0.67
Brangus[a,b]	32	0.19	0.81	32	0.56	0.44
Beefmaster[b]	8	0.00	1.00	8	0.25	0.75
Braford[b]	8	0.13	0.88	8	0.25	0.75
Bramousin[b]	4	0.75	0.25	4	0.75	0.25
Simbrah[b]	6	0.33	0.67	6	0.83	0.17
Total Crossbred	76	0.20	0.80	76	0.47	0.53
B. taurus						
Shorthorn[a,b]	38	0.08	0.92	38	0.08	0.92
US Holstein[c]	1380	0.43	0.57	1380	0.47	0.53
UK Holstein[d]	552	0.29	0.71	540	0.37	0.63
German Holstein[e]	160	0.33	0.67	160	0.39	0.61
German Fleckvieh[e]	120	0.31	0.69	120	0.57	0.43
German Brown[e]	82	0.56	0.44	82	0.84	0.16
Swiss Brown[e]	206	0.61	0.39	206	0.74	0.26
Swiss Scharzfleck[e]	52	0.52	0.48	52	0.58	0.42
Swiss Simmental x Red Holstein[e]	242	0.45	0.55	242	0.53	0.47
Japanese Holstein[f]	556	0.21	0.79	580	0.26	0.74
Japanese Black[f]	372	0.41	0.59	372	0.43	0.57
Polish Holstein[g]	562	0.37	0.63	562	0.46	0.54
Korean Holstein[h]	104	0.30	0.70	104	0.39	0.62
Other[b,i]	126	0.34	0.66	126	0.42	0.58
Total B. taurus	4552	0.38	0.62	4564	0.45	0.55

[a]This study, [b]Seabury et al., 2004a,b, [c]Brunelle et al., 2007, [d]Juling et al., 2006, [e]Haase et al., 2007, [f]Nakamitsu et al., 2006, [g]Czarnik et al., 2007, [h]Jeong et al., 2006, [i]Combination of 20 *B. taurus* breeds (see Methods for details)
