# Supplementary material for: Frequencies of polymorphisms associated with BSE resistance differ significantly between Bos taurus, Bos indicus, and composite cattle
Source: BMC Vet Res. 2008 Sep 22;4:36. doi: 10.1186/1746-6148-4-36 (PMC2569919; doi:10.1186/1746-6148-4-36)
Supplement: Additional file 2 — Genotype frequencies for the 23-bp and 12-bp insertion/deletion polymorphism for each breed. The 23-bp and 12-bp homozygous insertion (+/+), heterozygous (+/-), and homozygous deletion (-/-) genotype frequencies fro each breed of B. indicus, B. taurus, and B. indicus × B. taurus composite cattle. [file 1746-6148-4-36-S2.pdf]

Additional Table 2 - Genotype frequencies for the 23-bp and 12-bp insertion/deletion polymorphism for each breed

The 23-bp and 12-bp homozygous insertion (+/+), heterozygous (+/-), and homozygous deletion (-/-) genotype frequencies fro each breed of *B. indicus*, *B. taurus*, and *B. indicus* x *B. taurus* composite cattle.

	23 bp				12 bp			
	n	+/+	+/-	-/-	n	+/+	+/-	-/-
B. indicus								
Brahman[a,b]	30	0.03	0.30	0.67	30	0.90	0.10	0.00
Nelore[a,b]	14	0.00	0.00	1.00	14	0.86	0.14	0.00
Gir[a]	12	0.00	0.25	0.75	12	0.33	0.58	0.08
Guzerat[a]	1	0.00	0.00	1.00	1	0.00	1.00	0.00
Tabapua[a]	1	0.00	0.00	1.00	1	1.00	0.00	0.00
Total B. indicus	58	0.02	0.21	0.78	58	0.76	0.22	0.02
Crossbred								
Santa Gertrudis[a,b]	9	0.00	0.33	0.67	9	0.00	0.67	0.33
Brangus[a,b]	16	0.00	0.38	0.63	16	0.25	0.63	0.13
Beefmaster[b]	4	0.00	0.00	1.00	4	0.00	0.50	0.50
Braford[b]	4	0.00	0.25	0.75	4	0.00	0.50	0.50
Bramousin[b]	2	0.50	0.50	0.00	2	0.50	0.50	0.00
Simbrah[b]	3	0.00	0.67	0.33	3	0.67	0.33	0.00
Total Crossbred	38	0.03	0.34	0.63	38	0.18	0.58	0.24
B. taurus								
Shorthorn[a,b]	19	0.00	0.16	0.84	19	0.00	0.16	0.84
US Holstein[c]	690	0.18	0.51	0.31	690	0.23	0.50	0.28
UK Holstein[d]	276	0.05	0.49	0.46	270	0.11	0.52	0.37
German Holstein[e]	80	0.09	0.49	0.43	80	0.11	0.55	0.34
German Fleckvieh[e]	60	0.12	0.38	0.50	60	0.22	0.32	0.47
German Brown[e]	41	0.27	0.59	0.15	41	0.68	0.32	0.00
Swiss Brown[e]	103	0.41	0.41	0.18	103	0.57	0.40	0.03
Swiss Scharzfleck[e]	26	0.23	0.58	0.19	26	0.31	0.54	0.15
Swiss Simmental x Red Holstein[e]	121	0.21	0.48	0.31	121	0.31	0.45	0.25
Japanese Holstein[f]	278	0.05	0.32	0.63	290	0.07	0.38	0.55
Japanese Black[f]	186	0.10	0.61	0.28	186	0.13	0.61	0.26
Polish Holstein[g]	281	0.12	0.50	0.38	281	0.20	0.51	0.28
Korean Holstein[h]	52	0.12	0.37	0.52	52	0.20	0.41	0.39
Other[b,i]	63	0.12	0.44	0.44	63	0.21	0.42	0.36
Total B. taurus	2276	0.14	0.48	0.39	2282	0.20	0.48	0.32

[a]This study, [b]Seabury et al., 2004a,b, [c]Brunelle et al., 2007, [d]Juling et al., 2006, [e]Haase et al., 2007, [f]Nakamitsu et al., 2006, [g]Czarnik et al., 2007, [h]Jeong et al., 2006, [i]Combination of 20 *B. taurus* breeds (see Methods for details)
