# Supplementary material for: Frequencies of polymorphisms associated with BSE resistance differ significantly between Bos taurus, Bos indicus, and composite cattle
Source: BMC Vet Res. 2008 Sep 22;4:36. doi: 10.1186/1746-6148-4-36 (PMC2569919; doi:10.1186/1746-6148-4-36)
Supplement: Additional file 3 — Haplotype frequencies for the 23-bp and 12-bp insertion/deletion polymorphism for each breed. The 23-bp insertion-12-bp insertion (++), 23-bp deletion-12-bp insertion (-+), and 23-bp deletion-12-bp deletion (--) haplotype frequencies for each breed of B. indicus, B. taurus, and B. indicus × B. taurus composite cattle. [file 1746-6148-4-36-S3.pdf]

Additional Table 3 - Haplotype frequencies for the 23-bp and 12-bp insertion/deletion polymorphism for each breed

The 23-bp insertion-12-bp insertion (++), 23-bp deletion-12-bp insertion (-+), and 23-bp deletion-12-bp deletion (--) haplotype frequencies for each breed of *B. indicus*, *B. taurus*, and *B. indicus* x *B. taurus* composite cattle.

	n	++	-+	--
B. indicus				
Brahman[a,b]	60	0.18	0.77	0.05
Nelore[a,b]	28	0.00	0.93	0.07
Gir[a]	24	0.13	0.50	0.38
Guzerat[a]	2	0.00	0.50	0.50
Tabapua[a]	2	0.00	1.00	0.00
Total B. indicus	116	0.12	0.75	0.13
Crossbred				
Santa Gertrudis[a,b]	18	0.17	0.17	0.67
Brangus[a,b]	32	0.19	0.38	0.44
Beefmaster[b]	8	0.00	0.25	0.75
Braford[b]	8	0.13	0.13	0.75
Bramousin[b]	4	0.75	0.00	0.25
Simbrah[b]	6	0.33	0.50	0.17
Total Crossbred	76	0.20	0.28	0.53
B. taurus				
Shorthorn[a,b]	38	0.08	0.00	0.92
US Holstein[c]	1380	0.43	0.04	0.52
UK Holstein[d]	540	0.29	0.08	0.63
German Holstein[e]	160	0.33	0.06	0.61
German Fleckvieh[e]	120	0.31	0.07	0.63
German Brown[e]	82	0.56	0.28	0.16
Swiss Brown[e]	206	0.61	0.17	0.22
Swiss Scharzfleck[e]	52	0.52	0.06	0.42
Swiss Simmental x Red Holstein[e]	242	0.45	0.08	0.47
Polish Holstein[f]	281	0.35	0.11	0.52
Korean Holstein[g]	104	0.30	0.09	0.62
Other[b,h]	126	0.34	0.08	0.58
Total B. taurus	3604	0.40	0.08	0.53

[a]This study, [b]Seabury et al., 2004a,b, [c]Brunelle et al., 2007, [d]Juling et al., 2006, [e]Haase et al., 2007, [f]Czarnik et al., 2007, [g]Jeong et al., 2006, [h]Combination of 20 *B. taurus* breeds (see Methods for details)
